# Association between sleep apnea and ultrasound-defined liver fibrosis: Results from NHANES 2017 to 2020

**DOI:** 10.1097/MD.0000000000037949

**Published:** 2024-04-26

**Authors:** Zhi-Wei Zhao, Wen-Sen Huang, Ling Li, Li-Da Chen, Li Lin, Hui-Xue Zeng

**Affiliations:** a Department of Otolaryngology, Zhangzhou Affiliated Hospital of Fujian Medical University, Zhangzhou, Fujian Province, People’s Republic of China; b Department of Respiratory and Critical Care Medicine, Zhangzhou Affiliated Hospital of Fujian Medical University, Zhangzhou, Fujian Province, People’s Republic of China; c Department of Ultrasound, Zhangzhou Affiliated Hospital of Fujian Medical University, Zhangzhou, Fujian Province, People’s Republic of China.

**Keywords:** cross-sectional survey, liver fibrosis, NHANES, nonalcoholic fatty liver disease, sleep apnea

## Abstract

Liver fibrosis is a critical factor in the advancement of nonalcoholic fatty liver disease towards cirrhosis. There is limited research exploring the association between obstructive sleep apnea (OSA) and liver fibrosis among community populations. The present study aimed to assess the association between sleep apnea (SA) and liver fibrosis based on the National Health and Nutrition Examination Survey (NHANES). Data were acquired from NHANES survey cycle 2017 to 2020. We assessed liver fibrosis by the median values of liver stiffness measurement (LSM). The diagnosis of SA was based on participants’ response to sleep questionnaire. Univariate and multivariate logistic regression were used to validate the association of SA and liver fibrosis. A total of 7615 participants were included in this study. The LSM level of SA group was significantly higher than non-SA group. The proportion of liver fibrosis in SA group was markedly higher than that in non-SA group (14.0% vs 7.3%, *P* < .001). Univariate logistic analysis showed that SA was positively associated with liver fibrosis (OR = 2.068, 95%CI = 1.715–2.494, *P* < .001). Further multivariate logistic analysis revealed that SA was independently associated with increased risk of liver fibrosis after adjusting for confounding factors (OR = 1.277, 95%CI = 1.003–1.625, *P* = .048). The current study demonstrated an independent association between self-reported SA and increased risk of ultrasound-defined liver fibrosis in community-based sample.

## 1. Introduction

Obstructive sleep apnea (OSA), caused by the partial or complete collapse of the upper airways during sleep, contributes to characteristics such as snoring, sleep-related breathing pause, and even daytime sleepiness. OSA patients exhibit an elevated predisposition to conditions such as hypertension,^[1]^ metabolic dysregulation,^[2,3]^ cognitive impairment,^[4]^ cancer incidence,^[5]^ and involvement in motor vehicle accidents.^[6]^ The relationship between nonalcoholic fatty liver disease (NAFLD) and OSA is also gaining attention.^[7,8]^

Liver fibrosis is a progressive condition that is associated with NAFLD, representing a complex and advancing medical condition. The gold standard for assessing fibrosis is liver biopsy; however, its invasive nature and the associated risk of complications pose significant drawbacks. Moreover, due to the uneven distribution of histological lesions within the liver parenchyma, this results in sampling error. FibroScan® enables the noninvasive assessment of liver stiffness through the calculation of the propagation speed of an elastic shear wave induced by the transducer. This measurement correlates with liver stiffness and, consequently, provides valuable information about the degree of fibrosis.^[9,10]^

A previous investigation with small sample size based on clinical settings suggested that OSA was not associated with liver fibrosis assessed by acoustic radiation force impulse.^[11]^ However, another study demonstrated an independent relationship between self-reported OSA and histological liver fibrosis.^[12]^ So, the association between OSA and liver fibrosis is still in dispute. Furthermore, the majority of studies in this issue were conducted in a clinical context and constrained by small sample sizes. This study aimed to ascertain the association using FibroScan® with a large sample based on data from the National Health and Nutrition Examination Survey (NHANES).

## 2. Methods

### 2.1. Study population

NHANES, conducted by the Centers for Disease Control and Prevention, is a nationwide survey aimed at assessing the health and nutritional status of the American population. The ethics review board of the National Center for Health Statistics granted approval for all study protocols. Before data collection, written consent was obtained from each participant. In this study, data were acquired from survey cycle 2017 to 2020. Participants with complete data on exposures (code: SLQ040) and outcomes (liver stiffness measurement [LSM]) were included. The exclusion criteria included excess alcohol intake,^[13]^ pregnant women, age < 16 years. Participants with a history of viral hepatitis (positive hepatitis B surface antigen or hepatitis C virus RNA) were also excluded.

### 2.2. Outcome assessment

Vibration controlled transient elastography was conducted with a FibroScan model 502 V2 Touch (Echosens, Paris, France) by trained technicians. The examinations were conducted in accordance with the guidelines provided by the manufacturer. Examinations were deemed reliable only when a minimum of 10 LSM values were obtained following a fasting period of at least 3 hours, and the interquartile range/median was <30%. The threshold of LSM ≥ 8.2 kPa was set to indicate significant liver fibrosis.^[14]^ Other parameters including serum alanine aminotransferase (ALT), aspartate aminotransferase (AST), total bilirubin (TBIL), gamma glutamyl transpeptidase (GGT), total cholesterol (TC), triglycerides (TG), high density lipoprotein-cholesterol, low density lipoprotein-cholesterol (LDL-C) were also extracted.

### 2.3. Exposure assessment

The assessment of sleep apnea (SA) was conducted using the sleep questionnaire (code: SLQ040), which inquired about the frequency of snorting, gasping, or cessation of breathing while sleep.^[15]^ The determination of SA was contingent upon the subject responses to this questionnaire, categorized as never, rarely (1–2 nights per week), occasionally (3–4 nights per week), and frequently (5 or more nights per week). The presence of SA was considered for those reporting rarely, occasionally, or frequently.

### 2.4. Covariates

Based on previous research, potential confounding variables included age (years), sex (males or females), body mass index (BMI), race (Mexican American, other Hispanic, non-Hispanic white, non- Hispanic black, other races), family income-to-poverty ratio (PIR), smoking status (smokers and nonsmokers), hypertension, coronary heart disease (CHD) and diabetes mellitus. BMI was calculated using the formula: BMI = weight (kg)/height (m^2^). Smoking status was defined based on the question “Have you smoked at least 100 cigarettes in your lifetime?.” Hypertension, diabetes mellitus, and CHD were identified based on self-reported physician diagnoses.

### 2.5. Statistical analysis

We employed mobile examination center sample weights to address the intricate sampling design and non-response in NHANES. Continuous variables were reported as mean (standard deviation) for those with a normal distribution, and median (interquartile range) for those with a skewed distribution. Categorical variables were presented as counts (%). The differences between 2 groups were analyzed by the Mann–Whitney U-test for normally distributed data, and Student *t* test for normally distributed variables. Statistical comparison of categorical variables was conducted using the Chi-squared test. The exploration of relationships between various variables and liver fibrosis was conducted using univariate logistic regression methods. To assess the independent association between SA and liver fibrosis, multivariate logistic analysis was employed in 3 adjusted models. Model 1was adjusted for gender, age, BMI, and race. Model 2 was adjusted for gender, age, BMI, race, and smoking, PIR. Model 3 was further adjusted for diabetes, hypertension, and CHD. The missing values of confounders were managed using random forest interpolation with the “missForest” R package. Data calculations were performed using R software (version 4.3.0; R Foundation for Statistical Computing). A 2-sided *P* value <.05 was considered statistically significant.

## 3. Results

**Figure 1.**
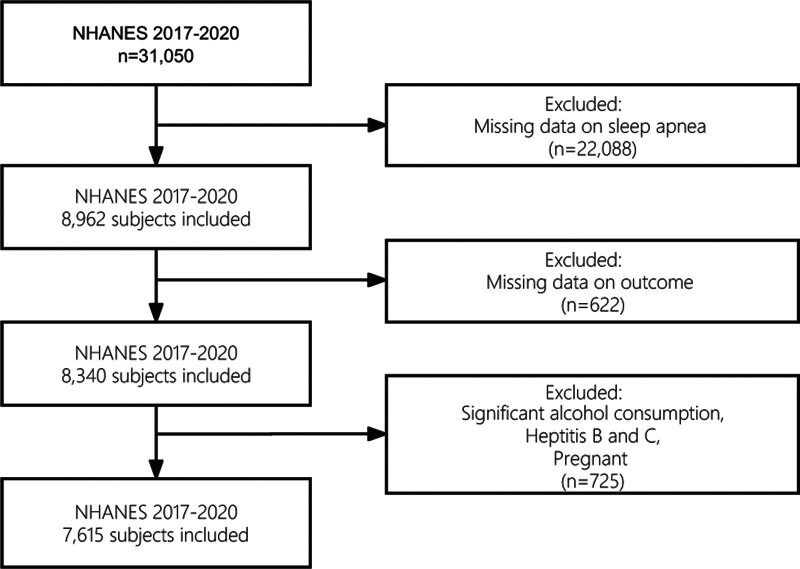
The flow chart of selection process.

We initially identified a total of 31,050 subjects. Following the exclusion of missing data in the SA questionnaire and outcome, 8340 participants were further included. At last, 7615 participants were included in this study after excluding individuals with significant alcohol consumption, viral hepatitis, and those who were pregnant (Fig. 1).

Table 1 displays the comparison of baseline characteristics between participants with and without SA. There were 1741 participants with SA and 5874 without SA. The racial distribution, PIR, TC, LDL-C and TBIL levels did not differ between the 2 groups. SA cases tended to be male, older. They also had higher BMI, TG, ALT, AST, GGT, higher rate of smoking, hypertension, diabetes, CHD. As for the marker of liver fibrosis, The LSM level of SA group was significantly higher than non-SA group. The proportion of liver fibrosis in SA group was markedly higher than that in non-SA group (14.0% vs 7.3%, *P* < .001).

**Table 1:** Baseline characteristics and liver fibrosis parameters in subjects with SA and without SA.

Characteristics	Overall	Non-SA	SA	*P* value
Number of subjects	7615	5874	1741	
Gender				<.001
Male, number (%)	3595 (46.7%)	2640 (44.2%)	955 (55.2%)	
Female, number (%)	4020 (53.3%)	3234 (55.8%)	786 (44.8%)	
Age, yr	46.33 ± 18.48	45.51 ± 18.88	49.18 ± 16.72	<.001
BMI, kg/m^2^	29.29 ± 7.29	28.64 ± 6.95	31.58 ± 7.99	<.001
Race				.474
Mexican American, number (%)	887 (8.4%)	703 (8.5%)	184 (7.8%)	
Other Hispanic, number (%)	759 (7.4%)	572 (7.1%)	187 (8.2%)	
Non-Hispanic White, number (%)	2577 (62.4%)	1957 (62.5%)	620 (62.1%)	
Non-Hispanic Black, number (%)	2030 (11.6%)	1557 (11.4%)	473 (12.1%)	
Other Race, number (%)	1362 (10.2%)	1085 (10.4%)	277 (9.8%)	
PIR	3.18 ± 1.63	3.18 ± 1.63	3.20 ± 1.65	.724
Smoking				<.001
Yes, number (%)	2664 (38.6%)	1912 (36.8%)	752 (44.7%)	
No, number (%)	4490 (61.4%)	3546 (63.2%)	944 (55.3%)	
Hypertension				<.001
Yes, number (%)	2610 (30.0%)	1819 (27.2%)	791 (39.9%)	
No, number (%)	4996 (70.0%)	4047 (72.8%)	949 (60.1%)	
Diabetes				.001
Yes, number (%)	1011 (10.2%)	694 (9.1%)	317 (13.7%)	
No, number (%)	6600 (89.8%)	5177 (90.9%)	1423 (86.3%)	
CHD				.038
Yes, number (%)	277 (3.8%)	182 (3.4%)	95 (5.0%)	
No, number (%)	6511 (96.2%)	4960 (96.6%)	1551 (95.0%)	
TG (mmol/L)	1.24 (0.87–1.82)	1.20 (0.85–1.75)	1.39 (0.97–2.06)	.006
TC (mmol/L)	4.81 ± 1.04	4.80 ± 1.05	4.82 ± 1.02	.673
HDL-C (mmol/L)	1.39 ± 0.40	1.42 ± 0.40	1.32 ± 0.38	<.001
LDL-C (mmol/L)	2.82 ± 0.91	2.82 ± 0.91	2.83 ± 0.89	.864
ALT (U/L)	17.00 (13.00–25.00)	17.00 (13.00–24.00)	20.00 (14.00–29.00)	.004
AST (U/L)	19.00 (16.00–23.00)	19.00 (16.00–23.00)	20.00 (16.00–24.00)	.005
GGT (U/L)	19.00 (13.00–28.00)	18.00 (13.00–27.00)	22.00 (15.00–33.00)	.002
TBIL (μmol/L)	6.84 (5.13–10.26)	6.84 (5.13–10.26)	6.84 (5.13–10.26)	.129
LSM (kPa)	4.90 (4.00–6.10)	4.80 (4.00–6.00)	5.10 (4.10–6.55)	<.001
Hepatic fibrosis				<.001
Yes, number (%)	743 (8.8%)	489 (7.3%)	254 (14.0%)	
No, number (%)	6872 (91.2%)	5385 (92.7%)	1487 (86.0%)	

All continuous variables and percentages for categorical variables were weighted, with the exception of the number of participants.

ALT = alanine aminotransferase, AST = aspartate aminotransferase, BMI = body mass index, CHD = coronary heart disease, GGT = gamma glutamyltransferase, HDL-C = high density lipoprotein-cholesterol, LDL-C = low density lipoprotein-cholesterol, LSM = liver stiffness measurement, PIR = family income-to-poverty ratio, SA = sleep apnea, TC = total cholesterol, TG = triglycerides, TBIL = total bilirubin.

Table 2 presents the results of univariate logistic analysis for liver fibrosis. The results revealed positive associations between liver fibrosis and variables such as age, male gender, BMI, the presence of hypertension, diabetes, CHD and SA. PIR was found to be negatively related with liver fibrosis. In addition, no relationship existed between liver fibrosis and smoking or race.

**Table 2:** The results of univariate logistic analysis for liver fibrosis.

	Liver fibrosis	*P* value
	OR (95% CI)	
Sex		
Female	1	
Male	1.662 (1.267–2.180)	<.001
Age	1.017 (1.012–1.023)	<.001
BMI	1.135 (1.118–1.152)	<.001
Race		
Mexican American	1	
Other Hispanic	0.939 (0.667–1.321)	.705
Non-Hispanic White	1.031 (0.689–1.541)	.877
Non-Hispanic Black	0.989 (0.707–1.383)	.945
Other Race	0.823 (0.564–1.199)	.294
PIR	0.913 (0.849–0.982)	.017
Smoking		
No	1	
Yes	1.245 (0.998–1.554)	.053
Hypertension		
No	1	
Yes	2.288 (1.748–2.995)	<.001
Diabetes		
No	1	
Yes	4.400 (3.430–5.645)	<.001
CHD		
No	1	
Yes	1.943 (1.351–2.793)	<.001
SA status		
No	1	
Yes	2.068 (1.715–2.494)	<.001

BMI = body mass index, CHD = coronary heart disease, OR = odds ratio, PIR = family income-to-poverty ratio, SA = sleep apnea.

Three adjusted models were applied for the interaction between SA and liver fibrosis. The results were summarized in Table 3. In a model adjusting for gender, age, BMI, and race, there was a significant association between SA and liver fibrosis (OR = 1.278, 95% CI = 1.018–1.603, *P* = .036). After further adjusting for other potential confounders (smoking and PIR), SA still significantly associated with liver fibrosis (OR = 1.292, 95% CI = 1.032–1.618, *P* = .028). After further adjusting for diabetes, hypertension, and CHD, the association remained significant (OR = 1.277, 95% CI = 1.003–1.625, *P* = .048).

**Table 3:** The multivariate logistic analysis for relationship between SA and liver fibrosis.

SA status	Model 1		Model 2		Model 3	
	OR (95% CI)	*P* value	OR (95% CI)	*P* value	OR (95% CI)	*P* value
No SA	1		1		1	
SA	1.278 (1.018–1.603)	.036	1.292 (1.032–1.618)	.028	1.277 (1.003–1.625)	.048

Model 1: adjusted for gender, age, BMI, and race.

Model 2: adjusted for gender, age, BMI, race, and smoking, PIR.

Model 3: adjusted for gender, age, BMI, race, smoking, PIR, diabetes, hypertension, and CHD.

BMI = body mass index, CHD = coronary heart disease, OR = odds ratio, PIR = family income-to-poverty ratio, SA = sleep apnea.

## 4. Discussion

In this retrospective study with a large sample size of community populations, the prevalence of ultrasound-defined liver fibrosis in the SA group was markedly higher than that in the non-SA group. Furthermore, following adjustments for multiple confounding factors, there was an independent association between SA and ultrasound-defined liver fibrosis.

The association between OSA and liver fibrosis is still far from conclusion. A study including 65 consecutive children with biopsy-proven NAFLD showed that the presence and severity of OSA were correlated with liver fibrosis, regardless of factors such as BMI, abdominal adiposity, metabolic syndrome, and insulin resistance.^[16]^ Another study enrolling 101 morbidly obese subjects suggested that liver fibrosis was notably more severe in highest oxygen desaturation index group. In multivariate analysis adjusting for age, obesity, and insulin resistance status, chronic intermittent hypoxia maintained an independent association with hepatic fibrosis.^[17]^ Lian et al^[18]^ reported that the severity of OSA emerged as an independent risk factor for blood markers of liver fibrosis in non-obese patients. However, some studies yield opposing conclusions. Trzepizur et al^[19]^ used FibroMeter NAFLD score to evaluate liver fibrosis and they found that the relationship between OSA severity and liver fibrosis was not maintained after adjusting for confounders. In addition, a study employed noninvasive blood test FibroTest to evaluate liver fibrosis in a cohort of OSA patients and revealed that no correlation was detected between liver fibrosis and nocturnal hypoxia.^[20]^

Previous studies were limited by a small sample size, leading to potentially unreliable conclusions. Moreover, the majority of research in this field was conducted in hospital settings, with limited representation from community-based studies. Recognizing these gaps, our study was designed to address these limitations and contributed to a more comprehensive understanding of the relationship between OSA and liver fibrosis. Our study demonstrated an independent association between SA and ultrasound-defined liver fibrosis in a large cohort of community populations.

The findings have significant clinical implications. This emphasizes the need for screening of the presence of OSA when evaluating and treating liver diseases, as this condition increases the risk of having liver fibrosis. Conversely, patients with NAFLD and newly diagnosed OSA should undergo evaluation for the presence of fibrosis. A research indicated that OSA patients who responded to continuous positive airway pressure treatment exhibited more pronounced improvement of lobular inflammation on liver biopsy than those who did not respond.^[21]^ Moreover, OSA treatment resulted in the decrease of circulating sCD163 and a trend toward an increase in sFasL.^[22]^ Both were markers of hepatocyte apoptosis and Kupffer cell activation. Given that liver fibrosis represents the advanced stages of liver disease, the potential cost-effectiveness of addressing OSA as part of a comprehensive treatment strategy becomes evident.

The mechanisms whereby OSA may contribute to liver fibrosis are multiple and include inflammation, oxidative stress and increased collagen expression.^[8,23]^ An animal study showed that mice subjected to CIH displayed observable fibrosis in the liver, but absent in the control group. CIH induced notable elevations in serum and liver tissue lipid peroxidation, along with elevated levels of proinflammatory cytokines and α_1_(I)-collagen mRNA.^[24]^

This study has certain limitations that warrant discussion. Firstly, the cross-sectional design limitation in the NHANES data hinders the verification of a causal association between SA and liver fibrosis. Secondly, polysomnography is considered the gold standard for diagnosing OSA, the identification of SA relied solely on participants’ responses to questionnaires, which potentially impacted the accuracy of the results. While self-reporting introduces the potential for recall bias, the NHANES questionnaire has been validated and widely used in epidemiological research, demonstrating acceptable sensitivity and specificity in identifying individuals with symptoms suggestive of SA. Thirdly, despite our efforts to include a comprehensive set of covariates in the study to account for potential influences in the model, there was still no guarantee that there were potential confounders leading to bias in the results. Finally, our study exclusively enrolled individuals aged 16 years or older. Consequently, the findings cannot be generalized to participants under 16 years old.

This study analyzed the data from the large-scale national US survey. Our findings demonstrated a significant association between self-reported SA and ultrasound-defined liver fibrosis after adjusting for confounders in community-based sample. Future prospective studies are necessary to clarify the causal relationship between OSA and liver fibrosis.

## Acknowledgments

We would like to thank all participants in this study.

## Author contributions

**Data curation:** Zhi-Wei Zhao.

**Formal analysis:** Wen-Sen Huang.

**Investigation:** Zhi-Wei Zhao.

**Methodology:** Ling Li, Li-Da Chen.

**Project administration:** Hui-Xue Zeng.

**Software:** Li Lin.

**Writing – original draft:** Zhi-Wei Zhao.

**Writing – review & editing:** Hui-Xue Zeng.
